# Analysis of the Actual One-Month Usage of Portable Listening Devices in College Students

**DOI:** 10.3390/ijerph18168550

**Published:** 2021-08-13

**Authors:** Gibbeum Kim, Jihun Shin, Changgeun Song, Woojae Han

**Affiliations:** 1 Department of Speech and Hearing Science, College of Applied Health Sciences, University of Illinois at Urbana-Champaign, Champaign, IL 61820, USA; gibbeum4528@gmail.com; 2 Laboratory of Hearing and Technology, Research Institute of Audiology and Speech Pathology, College of Natural Sciences, Hallym University, Chuncheon 24252, Korea; 3 Division of Speech Pathology and Audiology, College of Natural Sciences, Hallym University, Chuncheon 24252, Korea; 4 Department of Convergence Software, Graduate School, Hallym University, Chuncheon 24252, Korea; tho881@naver.com; 5 Department of Convergence Software, College of Information and Electronic Engineering, Hallym University, Chuncheon 24252, Korea; cgsong@hallym.ac.kr

**Keywords:** portable listening devices, noise-induced hearing loss, music exposure, tracking method, digital health, mobile application

## Abstract

Although contemporary researchers are concerned about overexposure of portable listening devices (PLD) for adolescents and young adults who often prefer listening to music at high levels for a long time, many of these studies have focused on either comparing sound pressure levels of various kinds of earphones or evaluating the recognition of noise-included hearing loss and listening habits through surveys. Further still, current criteria were developed for occupational noise-induced hearing loss, so there are only a few published guidelines for hearing insults due to recreational noise exposure. The present study, therefore, measures actual listening levels and PLD time in college students using a real-time measurement system and applying that gathered scientific data to the internationally recommended noise exposure standards. Thirty-four college students were asked to listen to music similar to their daily lifestyles for 4-weeks. After installing the application, the Google account that linked to the user’s mobile phone was logged into the server communication. When a subject listened to music, the average and maximum listening levels and listening time could then be recognized as his or her Google account ID and stored in the database for analysis. User data was measured at 1-s intervals and delivered to the main server system every 5 s. The data were analyzed as LZeq for mean levels and LCpeak for maximum levels, and also for PLD use time. The mean of the preferred listening level was 68–70 dB SPL for 4 weeks with long enough break times. That is, the listening levels of college students were not high enough to induce instant hearing loss when they used PLD. However, there was a large individual difference in the listening levels and use times. When applied to three recommended noise exposure criteria, the number of exceeded subjects also differed from 0 to 56.72% depending on the criterion. We thus suggest that appropriate and standardized criteria for music-induced hearing loss might be proposed for recreational PLD users.

## 1. Introduction

With the recent advances in technology and ever-increasing use of portable listening devices (PLDs), many researchers have warned of the potential risk of noise-induced hearing loss although there were users of Walkman and telephone headsets in the past. Since just 10 years ago, 90% of teenagers and college students have had PLDs, including MP3 players and iPods [1]. Smartphones with a free MP3 player function currently are one of several used PLDs types [2]. Such high accessibility to PLDs have made it easier to listen to music regardless of time and place [3,4], consequently producing unrestrained PLDs use by adolescents and/or college students in everyday life and causing decreased hearing sensitivity in this group [4,5].

Recommended exposure limits (RELs) have been used worldwide to prevent noise-induced hearing loss. In detail, the National Institute for Occupational Safety and Health (NIOSH) does apply the permissible noise exposure standard of 8 h per day at the intensity level of 85 dB in the workplace, which expresses 85 decibels A-weighted (dB[A]) as an 8-h time-weighted average (TWA) [6]. However, because it is the standard for employees who are being exposed to continuous machine noise in free-field environment (e.g., industrial sites), we claim that this is not an appropriate application for preventing hearing loss from recreational noise directly exposed to users’ eardrums through earphones used for listening to music [7]. In addition, many developed countries have applied an individual’s daily noise dose while using the rule of a 3 dB exchange rate with a level of 85 dB in terms of intensity and time of the noise exposure. In other words, a 100% daily noise dose is calculated by T(min) = 8 h × 60 min/2^(L−85)/3^, which is a formula that decreases the allowable time of noise exposure by 1/2 when the loudness of the sound increases by 3 dB [6,8,9]. Based on the results of these previous studies, the maximum outputs of PLDs volume exceeded 125 dBA in general [2,10], and the average listening level of the users was 71–105 dBA [11,12]. When putting these findings into the formula of the allowable listening time by NIOSH [6], a 100% daily noise exposure dose of 105 dBA allows for only 15 min of listening. Again, it is not a realistic and practical standard because the durations and frequencies of PLD usage vary greatly from person to person.

On the other hand, the World Health Organization (WHO) has suggested a 60–60 rule for PLDs users, which is a rule for listening for no more than 60 min a day at an intensity of less than 60% of the device’s maximum volume level [13]. Unfortunately, this standard is not a convincing one because the maximum volume level of PLDs varies by both manufacturers and models [2]. In sum, the recommended noise exposure standards are better for regulating the intensity and time from continuous noise exposure of workers in industrial fields but have a limitation as a regulatory standard used to prevent the increasing music-induced hearing loss of adolescents and young adults with various listening habits.

In general, contemporary studies have reported average use time of PLDs [14,15], conducted a survey to compare the users’ preferred volume levels and their self-awareness of the noise-induced hearing loss [16,17], measured the maximum volume outputs of PLDs [2,5] and investigated the correlation between earphone types and users’ volume outputs under artificial noisy conditions [18,19], rather than measuring listening time and levels (or intensity) daily. Although there were some consistent results for the subjective survey responses and the studies that physically measured the maximum output levels of PLDs at the in-ear of the users, these studies did not reflect everyday listening environments where various background noises exist, and there are individual listening patterns and habits. For instance, Hodgetts et al. [18] measured the listening levels from a microphone located in the subjects’ ear while listening to selected music in a laboratory setting, but they did not take into account the actual and realistic background situations to which users were exposed to daily. Haines et al. [20] tried to combine the survey responses of the users’ listening habits and their preferred listening levels in several laboratory settings. Although their results did show correspondence between the questionnaire and the actual preferred listening volume, they still did not reflect the different variables that can occur in everyday listening situations of PLD users.

The present study aims to closely monitor the listening levels and time for college students who use PLDs the most, while also tracking their listening pattern of long term for 4 weeks through an elaborately developed application, and then analyzing the levels of listening volumes depending on their life patterns. Finally, the findings can lay the groundwork for presenting realistic and actionable standards for PLD users by applying the collected scientific data to several internationally recommended noise exposure standards.

## 2. Materials and Methods

### 2.1. Participants

Considering the need for a minimum of 30 subjects based on the sample size calculation of the G-Power program [21], it was decided to have 34 native Korean adults (16 males and 18 females). They enrolled in University and possessing and using an Android mobile phone participated in this study. Their age range was 20 to 26 years old (mean age: 22.7 years, standard deviation: ±2.38). Without collecting any medical history, they were screened for evaluation by both a normal function of the middle ear (i.e., type A tympanogram) and normal hearing sensitivity with thresholds at less than 20 dB HL from 125 to 8000 Hz testing frequencies.

Before participating in the experiment, all subjects understood its purpose and procedures and signed a consent form. The research procedures and contents were approved by the Institutional Review Board (IRB) of Hallym University.

### 2.2. Development of a Mobile Phone Application

To measure actual listening level and time spent by the PLDs users exposed through their connected earphones when they were listening to music using the MP3 player on their mobile phones, an installable application for their own mobile phones was developed by Shin et al. [22].

**Figure 1:**
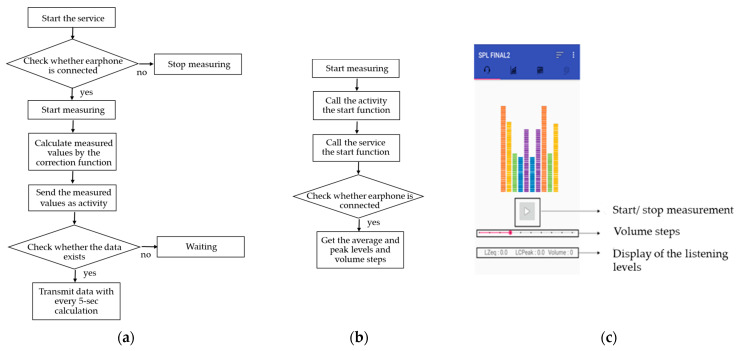
A flow chart showing the components of background service (**a**) and active processing (**b**), and a screenshot of the measurement application used in the current experiment using the developed application (**c**).

Figure 1 schematically shows both the internal and external functions of the measurement application. The application identifies the sound pressure levels of three noise situations for intensity. First, the ambient background noise level incoming through the microphone on the mobile phone was measured in real-time. Second, the Z-weighted equivalent sound pressure level or LZeq was measured and divided by the time needed for calculating the average listening level when a music file (e.g., mp3. or wav. file) was played through the earphone. Third, the highest sound pressure level between the start and end time of listening to the music was recorded as an LCpeak (C-weighted peak sound pressure level). The measured real-time data were stored as a database of the main server system every 5 s and analyzed by hourly/daily/weekly listening levels per individual user (Please see Shin et al. [22] for more detailed information regarding the application).

**Figure 2:**
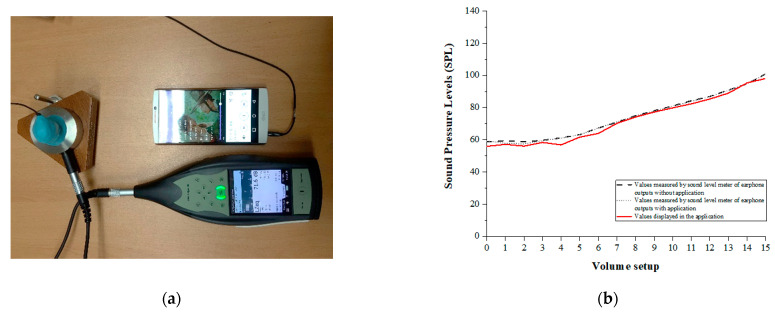
An example of sound pressure level measurement with an earphone connected to the application via a sound level meter and artificial ear simulator (**a**). The results of consistent level increasing with an increase of the volume steps in three measurement methods (**b**).

Before beginning the experiment, the accuracy of the noise levels measured through the application was confirmed using a sound level meter (Type #2250, Bruel & Kjær, Nærum, Denmark) and an artificial ear simulator (Type #4153, Bruel & Kjær, Nærum, Denmark) while randomly selecting and presenting two different types of music (i.e., ballad and dance) (Figure 2a). Further, to minimize any technical bias due to a difference in earphone types and models, the intensity at each volume level was analyzed through one designated earphone (Xiaomi PISTON 3, Beijing, China) distributed to the subjects during the experiment. That is, as the volume was increased by one step in the mobile phone to a total of 16 steps (0 to 15 volumes), the intensity of the sound outputs was presented in the system connected to the earphone while the application was running. The average of the sound pressure intensity (LZeq) and the maximum sound intensity (LCpeak) of the songs were matched within ±5 dB, while also confirming the accuracy and reliability of the music application (Figure 2b).

### 2.3. Experiment Procedures

The subjects who passed the hearing screening tests installed the developed application on their own Android mobile phone. They were asked to use the same type of earphone (Xiaomi PISTON 3, Xiaomi, Beijing, China) that had been pre-checked for the accuracy of the application. During the 4-week experiment, they listened to music through the earphone according to each individual’s life pattern and their usual listening patterns. However, they were asked to write a note if they were experiencing an unusual life pattern (i.e., visiting a quiet library, staying in a noisy gym, etc.).

After installing the application, the Google account already linked to the user’s mobile phone was logged into the server communication. When a subject listened to music, the average and maximum listening levels and listening times were recognized as that person’s Google account IDs and stored in the database for analysis. Users’ data were measured at 1-s intervals and then delivered to the main server system every 5 s (Figure 1a).

### 2.4. Data Analysis

The listening levels and time of individuals that transmitted to the server system from the application were analyzed daily. The average listening levels and peak levels that accumulated over 4 weeks were also explored on a weekly and monthly basis for each subject. The experimental period was the same for all participants at a total of 28 days, but the start date was slightly different for each subject. Thus, when analyzing the listening level for each week, the average listening level (LZeq) and the maximum listening level (LCpeak) were analyzed for 7 days (a full week) based on each individual’s start date.

Statistical analysis was performed by using SPSS software (ver. 23.0, IMB Co., Armonk, NY, USA). One-way analysis of variance (ANOVA) with a repeated measure was performed to compare average listening levels and the highest listening levels for 4 weeks. If necessary, a Bonferroni post-hoc analysis was applied to confirm the meaningful relationship in greater detail. The criteria for significance level was *p* < 0.05.

## 3. Results

### 3.1. Analysis of Listening Levels

The group mean for the average listening levels and the highest listening levels was 69.33 ± 5.50 dB SPL and 114.24 ± 6.66 dB SPL for 4 weeks, respectively. Table 1 displays the individual data of the listening levels (LZeq and LCpeak) and listening duration (on a daily and monthly basis). Among the 34 subjects, one who listened to the music at the lowest intensity was noted at 57.32 dB SPL (Sub 22 in Table 1), and the person who listened to it at the highest intensity was noted at 83.43 dB SPL (Sub 19). The minimum of LCpeak was 94.71 dB SPL (Sub 18), while the maximum was 121.5 dB SPL which seemed to be a very high value (Sub 15).

**Figure 3:**
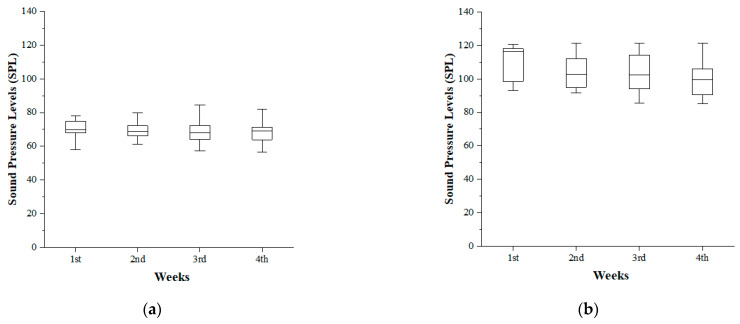
Box-and-whisker plot of mean preferred listening levels for 4 weeks: Average Z-weighted equivalent sound pressure levels (LZeq) (**a**) and maximum C-weighted peak sound level (LCpeak) (**b**).

In Figure 3, the first week shows the highest average listening level with 71.1 ± 5.36 dB SPL for the four weeks, and the second, third, and fourth weeks listening to music at the intensity of 69.26 ± 5.63 dB SPL, 68.53 ± 5.96 dB SPL, and 68.17 ± 6.22 dB SPL, respectively (Figure 3a). Statistically, one-way ANOVA with repeated measures showed a significant difference between the weeks for the average listening level [F(3,99) = 7.98, *p* = 0.000]. As a result of a Bonferroni post-hoc analysis, there were statistically significant differences between the first week and the second to fourth weeks (*p* < 0.05), indicating that subjects had consistently listened to PLDs with an average of 68–70 dB SPL between second to fourth weeks unlike the first week when seemed to be conscious of participation in the experiment.

The maximum listening level was a similar pattern to the average listening level, having the highest intensity at 110.60 ± 10.10 dB SPL in the first week, 103.48 ± 9.25 dB SPL in the second week, 103.11 ± 10.90 dB SPL in the third week, and 100.87 ± 10.92 dB SPL in the fourth week (Figure 3b). The result of statistical analysis indicated there was a significant difference between the weeks [F(3,99) = 7.545, *p* = 0.000]. The post-hoc analysis produced a statistically significant difference between the first week and the second to fourth weeks (*p* < 0.05) while confirming the same pattern as the average listening level and showing the highest listening levels with an average of 100–103 dB SPL.

### 3.2. Analysis of Listening Duration

#### 3.2.1. Daily Listening Time

The average daily listening time for the 34 subjects during the 4-week experimental period was about 140 min (or 2.3 h). 56.72% of subjects listened to music for more than 1 h per day. In Table 1, the listening time of PLDs showed great variance for subjects who listened for as short a time as 46 min per day (See the Sub 28 in Table 1) to one who listened for as long as 324 min (about 5.5 h; Sub 31 in Table 1). During the study period, the number of days that the subjects listened to PLDs ranged widely from as little as 12 days to as many as 28 days, indicating that several persons had actually listened every day for the full 4 weeks. Additionally, Friday and Tuesday had significantly the highest (71.08 dB SPL) and the lowest (67.42 dB SPL) levels, respectively.

#### 3.2.2. Continuation and Break Time for Listening

The continuous listening time and break (or rest) time were analyzed for 4 weeks. When looking at them roughly, 32 out of 34 subjects (94.12%) listened to music continuously for 2 h or more per day. Most of the subjects listened to music for more than 3 days out of 28 days with continuous 3 to 4 h of listening time. On closer analysis, it was divided into three groups: one group for less than 1 time with continuous 2-h listening, one for 3 or more times with continuous 3 to 4-h listening, and the other for 3 or more times with continuous 4-h of listening. These groups included 2, 24, and 8 subjects, respectively. In other words, our participants did not listen for as long as we thought, and their listening duration was not a significant problem with hearing loss.

Fortunately, it was also confirmed that all of the subjects had long enough breaks after their continuous listening time. For example, after listening to music for up to 5 h continuously, they took a break of about 6 h or more. Further, even if some of the subjects listened to music continuously, they took a longer break than their listening time. Contrary to our concern, several participants did not listen to music every day and sometimes even had days when they did not listen to music all for the day.

### 3.3. Applicative Analysis by International Recommended Exposure Limits (RELs)

#### 3.3.1. REL 1: 8 h/85 dB(A) (NIOSH, 1998)

Based on the start date for each subject, the listening time of 28 days (4 weeks) was analyzed by applying the exposure limit of 85 dB level for 8 h a day. Simply put, none of the 34 subjects (0%) listened to music in excess of the REL of NIOSH (1998) who listened to more than 8 h a day at the intensity level of 85 dB or higher. To analyze NIOSH’s recommended intensity or time, our participants’ use as recalculated by taking the total number of participation days for the 34 subjects (28 days × 34 persons = 952 cases; See Table 2). In other words, as a result of analyzing each hour by dividing that individual’s daily listening time into 480 min (60 min × 8 h) or less than 60 min to 480 min or more, the number of subjects (413 cases) who listened for less than 60 min a day was the most common instance at 43.38%.

#### 3.3.2. REL 2: Formula of T(min) = 480/2 ^(L−85)/3^ (NIOSH; OSHA; American Academy of Audiology, 2003)

The allowable noise exposure time was analyzed based on the daily exposure intensity of 34 subjects. When converting 24 h a day into minutes (24 h × 60 min), 9 subjects (26.4%) listened to music for less than 1440 min during the 28-day experimental period. Individual data was applied to REL 2, and 23.40% (223 out of 952 cases) exceeded the recommended exposure limit. However, as we expected, a large individual variance was found. Some subjects who listened to the music at the lowest intensity (57.32 dB SPL) were allowed to listen to music for 1424 min (or 23.7 h) whereas those subjects who listened with the intensity of 98.38 dB SPL had an allowable exposure time of only 22 min.

#### 3.3.3. REL 3: 60–60 Rule (WHO, 2015)

To apply the WHO 60–60 recommended exposure standard, the volume of 1 to 15 steps was analyzed using three mobile phone models (Samsung Galaxy S6, LG G3, and Samsung Note 4) that are currently marketed and used the most. At the maximum volume level, 98.71 ± 1.99 dB SPL, the average volume intensity of the three models, was calculated as an intensity equivalent to 60% of the maximum volume, which is 98.71 × 0.6 = 59.22 dB SPL. Then, the sum of the total listening volume for 4 weeks (28 days) was calculated and analyzed to determine the listening volume per day. When applied to the formula, 56.72% (540 out of 952 cases) listened to music at an intensity of 59.22 dB SPL or higher for 60 min or more per day.

## 4. Discussion

To overcome the limitations of studies that have measured recognition on hearing loss using subjective questionnaires and studies that have measured preferred listening levels in an artificially manipulated environment [2,5,14,15,16,17,18,19], the present study analyzed the real-time data of actual listening volume intensity and use the time while listening to music, while at the same naturally reflecting life patterns of college students. We also compared our data to three recommended acceptable noise exposure standards, which determine the appropriateness of the criteria for preventing general noise-induced hearing loss to PLD users and their voluntary and recreational purposes and use.

### 4.1. Are the Listening Levels and Listening Times of College Students Using PLD Risky Enough to Cause Hearing Loss?

Most previous studies have reported that PLDs users consistently use PLDs for more than 8 h a day at an intensity of 85 dBA, and have warned against the possibility of noise-induced hearing loss [10,23]. However, our one-month tracking study showed that the average listening volume intensity for 4 weeks was 69.33 dB, and not even one subject exceeded 85 dB, the permissible noise exposure level. It thus can be seen that PDLs users who listen to music in actual daily life do not listen to music at the high intensity that causes noise-induced hearing loss. Nevertheless, the possibility of hidden hearing loss which indicates that cochlear neuropathies can appear at lower exposure levels and is not well represented in typical audiological testing should be taken into account [24].

In a previously reported survey by Danhauer et al. [14], the average listening time of college students per day was reported to be 30~60 min. As confirmed in our own experiments, the number of subjects who listened to music for less than 60 min per day was the highest number. However, since the use of PLDs does demonstrate a large difference based on individual characteristics and life patterns, not only the listening sound level but also the listening time, should be considered as an index for evaluating their possible risk for noise-induced hearing loss.

Interestingly, most of the subjects had an appropriate rest time (about 6 h) after listening to music continuously. Subjects took a rest period of about 6 h after maximum noise exposure, which indicates that even if they were exposed to excessive noise levels, the risk of noise-induced hearing loss was greatly reduced when appropriate behavioral correction is made [25]. In this way, even in a survey study of college students, more than half of the respondents sought out a warning function in their mobile phones when listening to a volume above an appropriate listening volume level as their solution to excessive listening to music with a high volume that can cause hearing loss [14].

### 4.2. Are the Currently Internationally Proposed Standards for Noise-Induced Hearing Loss Sufficiently Applicable to PLD Users?

Several studies have been conducted to establish recommended standards for preventing noise-induced hearing loss. Of the three allowable noise exposure standards used internationally, our experiment determined that the number of subjects exceeding the WHO 60–60 rule limiting enjoyable noise exposure was more than half (56.72%). As mentioned in our introduction, considering that less than half of the number of college students follow the WHO 60–60 rule, it can be said that the currently proposed standard is a very unrealistic prevention proposal.

On the other hand, our study showed that no subject exceeded the criterion (0%) when it was applied to the NIOSH standard although we only used music exposure from the PLDs without considering other sources of music, professional or leisure exposure. This result is very similar to the results of Worthington et al.’s study [26], which indicated that only about 4% of their subjects exceeded the NIOSH 1998 standard in a noisy situation in a questionnaire survey of their usage time. When applied to the other standard offered by NIOSH, OHSA, or AAA, about 23% of our study subjects exceeded it. We thus need to seriously think about which criterion is the most appropriate. At first glance, it can be said that the WHO standard seems to be the strictest, and the NIOSH 1998 standard, which is the same as the prevention standard for occupational noise-induced hearing loss, looks to be the most tolerant to the PLD users. However, the strict standards that do not consider the reality and life pattern of PLD users, or standards that are applied to workers who listen to and work with continuous machine noise every day, are not appropriate enough to prevent hearing loss for today’s rapidly increasing PLD users. In conclusion, rather than suggesting the currently proposed uniform prevention standard for noise-induced hearing loss to PLD users, it would be desirable to monitor individual’s listening patterns including preferred listening levels and use time to recommend a customized preventive solution. It can be used like a digital health device currently applied in the market, such as heart rate checking and walking/running checking tools.

### 4.3. Limitations and Further Study

There were certain limitations to this that warrant further study. First, unlike previous studies, which applied A-weighted [18], the preferred listening volume was measured in this study using the application to which the dB SPL was applied because of the limited technology of the developed application. Thus, it will be necessary for the future to further supplement the technique for applying the A-weighted value for each frequency to the listening volume application and measure the accurate average listening levels and the highest listening levels based on dB A [27], consequently providing a meaningful relationship between preferred listening levels of PLD users and levels and type of background noise and informing a detailed profile of background noise to which PLD users are exposed in real life by further study

Secondly, there was a significant difference between the preferred listening levels on the earphones in the study conducted by Fligor and Ives [28]. However, we limitedly used only one particular type of earphone for all participants to secure greater reliability of results, but without considering individual preference for an earphone. In any follow-up study, it should be confirmed that different types of earphones can be measured by utilizing various types of earphones, such as earbuds, on the ears, and headsets. Such a change could scientifically explain more of the natural life patterns of young PLD users more accurately. Finally, although the results of the present study are long tracking experimental data for 4 weeks, the sample size (n = 34) is insufficient to generalize our results. In the following study, it is recommended to experiment with a larger number of PLD users with various characteristics and to analyze the results from several prospective.

## 5. Conclusions

After closely monitoring the listening levels and time for 34 college students while tracking their listening pattern of long term for 4 weeks by using the application, we found that their listening levels were not high enough and their listening time were not very long to induce instant hearing loss when using the PLD. However, there was a large individual difference in the listening levels and use times. When applied to three recommended noise exposure criteria, the number of exceeded subjects also differed from 0 to 56.72% depending on the criterion. We suggest that appropriate and standardized criteria for music-induced hearing loss for recreational PLD users might be proposed while conducting further following studies.

## Figures and Tables

**Table 1:** Individual data for listening levels and times measured for four weeks (N = 34).

	Listening Levels		Listening Duration	
Subjects	LZeq (dB SPL)	LCpeak (dB SPL)	Average Time per a Day (min)	Average Day per a Month (Day)
Sub 1	76.80	117.48	71	25
Sub 2	74.63	117.28	141	24
Sub 3	70.40	113.85	142	22
Sub 4	71.56	115.99	54	25
Sub 5	67.76	117.68	84	26
Sub 6	67.49	116.92	93	12
Sub 7	66.97	120.43	177	16
Sub 8	69.07	118.34	170	21
Sub 9	62.97	103.00	110	17
Sub 10	64.72	117.66	196	27
Sub 11	71.30	114.17	276	22
Sub 12	65.17	108.75	74	24
Sub 13	66.56	101.67	152	17
Sub 14	72.58	120.74	101	27
Sub 15	79.91	121.50	107	23
Sub 16	67.61	117.45	151	18
Sub 17	61.94	114.47	87	22
Sub 18	73.01	94.71	275	19
Sub 19	83.43	107.35	181	20
Sub 20	66.07	116.59	138	25
Sub 21	69.95	118.53	164	28
Sub 22	57.32	115.28	132	27
Sub 23	62.21	114.07	87	28
Sub 24	66.45	103.48	189	28
Sub 25	69.83	117.26	113	26
Sub 26	66.31	118.09	126	20
Sub 27	65.14	120.04	122	27
Sub 28	67.48	114.01	46	19
Sub 29	67.27	117.95	133	26
Sub 30	70.56	118.48	87	27
Sub 31	80.70	120.60	324	22
Sub 32	69.08	103.78	218	22
Sub 33	69.90	105.39	143	21
Sub 34	75.28	121.26	74	25
Mean	69.33	114.24	140	23
SD	±5.50	±6.66	±64	±4

Sub: Subject; LZeq: Z-weighted equivalent sound pressure level; LCpeak: C-weighted peak sound pressure level; SD: standard deviation.

**Table 2:** Number and percentage of subjects for a sub-group of listening levels and time (N = 952).

Variable	Number of Users (%)	Range (Min to Max)	Mean (SD)
Listening time (min)			
<60	413 (43.38%)	0–59	31 (±14)
60–120	183 (19.22%)	63–119	93 (±34)
120–180	145 (15.23%)	121–179	152 (±50)
180–240	82 (8.61%)	181–239	217 (±55)
240–300	53 (5.57%)	241–299	265 (±52)
300–360	39 (4.10%)	301–359	333 (±60)
360–420	21 (2.20%)	361–419	385 (±45)
420–480	0	-	-
>480	16 (1.68%)	481–1438	700 (±90)
Listening level (dB SPL)			
<55	169 (17.75%)	48.75–54.89	49.97 (±5.9)
55–65	212 (22.27%)	55.5–64.51	60.67 (±2.95)
65–75	390 (40.97%)	65.09–74.68	69.87 (±4.2)
75–85	162 (17.02%)	75.02–84.79	78.24 (±5.4)
>85	19 (1.99%)	86.56–98.38	89.26 (±10.3)

## Data Availability

Not applicable.
